# Measurement of Hand Function by an Automated Device—System Validation and Usability Analysis

**DOI:** 10.3390/s25227068

**Published:** 2025-11-19

**Authors:** Margarida Vieira, Tobias Barth, Matthias Münch, Natascha Koch, Alexander Kögel, Marie Stroetmann, Arndt Peter Schulz

**Affiliations:** 1 NOVA School of Science and Technology, Department of Physics, 2829-516 Caparica, Portugal; 2 BG Klinikum Hamburg, Centre for Clinical Research, 21033 Hamburg, Germany; t.barth@bgk-hamburg.de (T.B.); m.muench@bgk-hamburg.de (M.M.); schulz@biomechatronics.de (A.P.S.); 3 German Research Center for Artificial Intelligence (DFKI), 23538 Lübeck, Germany; natascha.koch@dfki.de; 4 BG Klinikum Hamburg, Hand Rehabilitation Department, 21033 Hamburg, Germany; a.koegel@bgk-hamburg.de (A.K.); m.stroetmann@bgk-hamburg.de (M.S.); 5 Medical Faculty, Universität zu Lübeck, 23538 Lübeck, Germany; 6 Fraunhofer IMTE, Fraunhofer Research Institution for Individualized and Cell-Based Medical Engineering, 23538 Lübeck, Germany

**Keywords:** range of motion, hand mobility, IMU, sensors, physiotherapy, goniometer, IR camera

## Abstract

(1) Aim: This study aims to assess the repeatability and accuracy of a 9-axis IMU-based glove and an IR camera system, in order to determine their potential to replace traditional goniometry. (2) Background: Traditional methods for assessing hand function, such as goniometry, are time-consuming and limited by subjectivity, inter-rater variability and external factors that compromise accuracy and reliability. Recent advancements in motion capture technology and sensor-based devices offer potential improvements in efficiency and accuracy for hand rehabilitation assessment; (3) Methods: To evaluate the repeatability of an IMU-based glove and an IR camera, measurements were taken using a silicone hand model under controlled conditions, while accuracy assessments involved a volunteer without movement constraints. Bland–Altman plots were employed for visual comparison and accuracy evaluation; (4) Results: The Nuada glove exhibited high repeatability, with standard deviations below two degrees across all joints, surpassing the goniometer’s accuracy threshold of five degrees. The UltraLeap system demonstrated comparable repeatability, with deviations consistently under 3.5 degrees. Accuracy assessments revealed limitations: over 50% of the Nuada glove’s measurements and over 80% of UltraLeap’s measurements deviated by more than five degrees compared to the goniometer. However, the Nuada glove and UltraLeap system were more consistent with each other than with the goniometer, suggesting limitations in the goniometer’s reliability for modern mobility assessment; (5) Conclusions: Both devices exhibited excellent repeatability, highlighting their strong potential for clinical application. However, their accuracy compared to the goniometer requires further refinement. These findings suggest that these technologies could enhance traditional assessment methods, offering more efficient and accurate solutions for evaluating hand mobility in clinical settings.

## 1. Introduction

Hand therapy plays a vital role in rehabilitation, where assessing joint range of motion (ROM) is a fundamental component of diagnosis and treatment planning [1]. Conventional methods for evaluating hand function often depend on subjective assessments, which are insufficiently sensitive to detect subtle changes in hand mobility [2]. While goniometers are commonly used in clinical practice due to their simplicity, they are prone to inherent inaccuracies and influenced by external factors, which can undermine their reliability and clinical effectiveness [3]. Previous work on this topic has also demonstrated a high interrater variability, further emphasizing the challenges associated with traditional ROM measurement techniques [4]. Variability in measurements arises from factors such as the application method and patient anatomy, presenting significant challenges to precise ROM measurements [5]. Joints do not have fixed axes of motion due to sliding and rotational movements, making it difficult for goniometers to capture exact angular displacements. Further complicating matters are differences in arm length among goniometer designs, which can influence accuracy, while finger interference and tool misalignment were cited as potential contributors to these inconsistencies [6].

Another critical issue is inter-rater variability among therapists. Measurements often vary between therapists or even the same therapist at different times [7]. Studies highlight that inter-rater variation is generally greater than intra-rater variation, especially for lower extremity motions [8]. To achieve reliable results, standardization in testing methods is crucial. Nonetheless, even when standardized procedures are adopted, discrepancies of up to seven degrees have been reported between goniometric readings and gold-standard imaging techniques like radiography [9].

These challenges highlight the urgent need for innovative solutions in hand mobility assessment. Advanced technologies, such as sensor-based and motion-capture systems, offer more reliable and efficient alternatives. These systems are designed to provide more objective measurements, improving usability and therapists’ effectiveness, reducing the time effort and leading to better rehabilitation outcomes, ultimately enhancing patients’ quality of life [2].

## 2. Literature Review

In recent years, technological advances have led to the development of several sensor-based and motion-capture systems for hand function assessment and rehabilitation. Among these, IMU-based gloves and IR camera systems have shown strong potential to overcome the limitations of conventional goniometry, which is time-consuming, subjective and prone to inter-rater variability [3,4,5,6,7,8,9].

**Figure 1:**
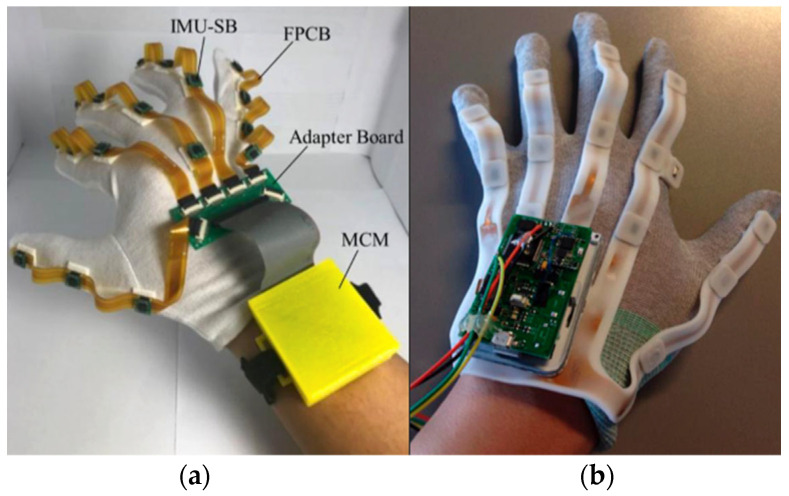
(**a**) Bor-Shing Lin et al. prototype [10]; (**b**) Fei Fei et al. glove prototype [11].

Several modular IMU-based data gloves have been proposed, such as those developed by Bor-Shing Lin et al. [10] and Fei Fei et al. [11], shown in Figure 1. Lin’s glove integrates 18 nine-axis IMUs capable of tracking joint movements with a mean angle error of ±3°, while Fei Fei et al. reported deviations of approximately 1.4° compared with goniometric measurements, both within clinically acceptable limits. However, these systems still face significant challenges, including sensor drift, magnetic interference, calibration dependence and limited usability in clinical environments. In contrast, the Nuada glove used in this study was designed to address some of these practical issues, focusing on wearability, ease of use and integration into clinical workflows, while maintaining the measurement precision required for medical applications.

IR systems, such as the Leap Motion Controller (LMC) and the UltraLeap, have been investigated as non-contact alternatives for hand motion tracking [12,13]. These systems provide accurate and comfortable assessment by eliminating the need for physical attachments. Studies by Gonçalves et al. [14] and others have demonstrated promising accuracy under static conditions; however, their performance decreases with dynamic movements or finger overlap due to occlusion and tracking instability. Although several studies have explored UltraLeap’s capabilities, most have focused on software or algorithmic validation rather than a direct comparison with standard clinical tools.

Therefore, the present study builds upon and complements previous work by conducting a systematic comparative evaluation of a 9-axis IMU-based glove (Nuada) and the UltraLeap IR camera system. By examining repeatability and accuracy—the two fundamental metrics required for clinical acceptance—under both controlled and realistic conditions, this research aims to bridge the gap between engineering validation and clinical applicability, demonstrating how these technologies can address existing limitations and contribute to more efficient and reliable hand mobility assessment. Repeatability refers to the consistency of an instrument under identical conditions [15], indicated by the standard deviation, while accuracy measures how close a measurement is to the true value [16]. These parameters are assessed using the Bland–Altman method, which evaluates the level of agreement between two measurement techniques [16,17].

## 3. Materials and Methods

The setting of this study was conducted in the Biomechanical Research Department, in cooperation with the Department of Hand Rehabilitation, at BG Klinikum, Hamburg, Germany, a large hospital specializing in musculoskeletal trauma.

### 3.1. Materials

#### 3.1.1. Goniometer

**Figure 2:**
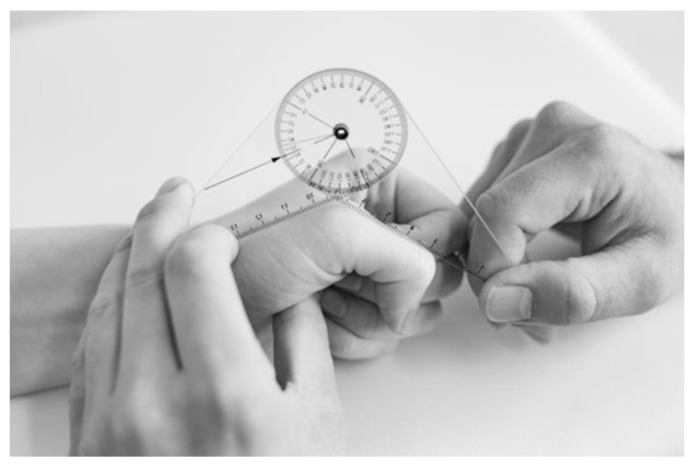
Short-arm goniometer.

This instrument, widely used in clinical settings, is considered the gold standard for assessing patients’ ROM with a technical accuracy of five degrees [18]. It consists of a short-arm goniometer, as seen in Figure 2, with a resolution of five-degree increments. It is usual practice to use only five or even 10-degree increments when assessing and reporting patients’ hand and finger movements.

#### 3.1.2. Silicone Hand Model

To ensure consistent and reliable measurements across various instruments, a silicone hand model was developed. This model was based on the right hand of a 23-year-old healthy female with no history of hand injuries. This approach aimed to maintain consistent coordinates and angle values when using multiple ROM measurement instruments. The following materials were used for the silicone models: Moulding Alginate (Michelangelo, Algaplay, Moncalieri, Italy) and silicone (ELASTOSIL ^®^ M 4644 A/B, Wacker Chemie AG, München, Germany, [19]).

#### 3.1.3. Nuada Glove

**Figure 3:**
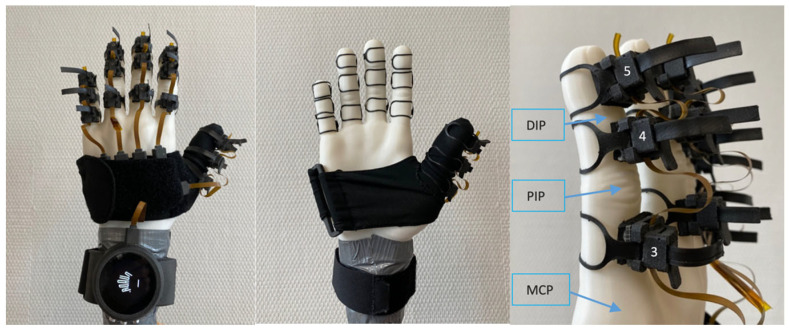
Nuada Glove prototype.

The Nuada measurement glove (prototype, NUADA, Porto, Portugal), represented in Figure 3, was developed by the Portuguese startup Nuada to facilitate the measurement of hand mobility. It features 18 nine-axis IMU sensors (BNO055 Intelligent 9-axis absolute orientation sensor [20], Bosch Sensortec GmbH, Reutlingen, Germany), strategically placed on each phalanx of the five fingers, as well as on the hand and wrist. Each sensor integrates an accelerometer, magnetometer, and gyroscope, employing a proprietary fusion algorithm to determine its orientation, which is provided as either quaternion data or Euler angles (roll, pitch, and yaw). To calculate a joint’s angle, the system uses both the sensor before and the sensor after the joint. For example, in Figure 3, the distal interphalangeal (DIP) joint angle detection relies on sensors four and five, the proximal interphalangeal (PIP) joint angle detection uses sensors three and four, while the metacarpophalangeal joint (MCP) depends on sensor three and sensor placed on the palm. Since several joints share the same sensor inputs, improper calibration of a single sensor can compromise multiple joint measurements.

Unlike traditional textile gloves, the Nuada glove uses a ring-based system to securely attach sensors to specific phalanges, improving accuracy and ease of wear, especially for users with hand limitations, designed to fit custom hand sizes by adjusting the rings. Its modular design features five flexes, each embedded with three sensors, connected to a palm-mounted board and a wrist unit via USB-C. This wrist unit, designed in the form of a watch, transmits data at 50 Hz via Bluetooth to a mobile application.

A hard calibration is needed to align the accelerometer, gyroscope, and magnetometer by positioning the accelerometer in multiple orientations, keeping the glove static for the gyroscope, and performing an eight-shaped movement for the magnetometer. After this, only the magnetometer requires recalibration with the same movement at each restart due to its sensitivity to environmental magnetic fields [21].

#### 3.1.4. UltraLeap Motion Controller 2

**Figure 4:**
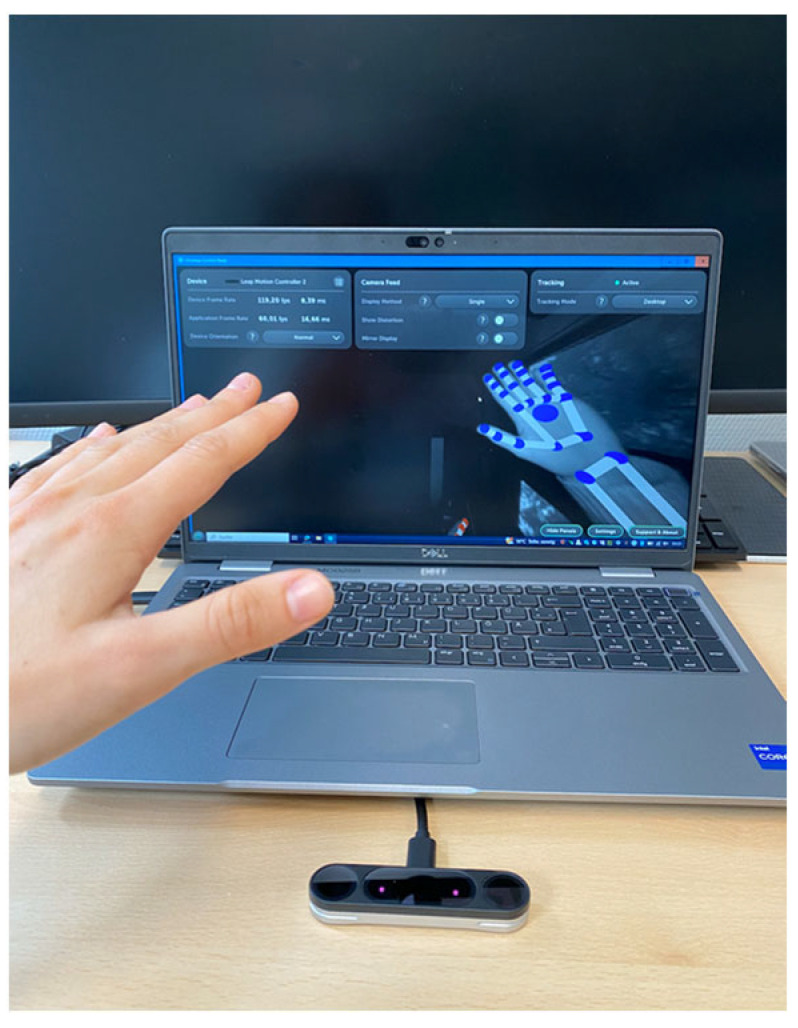
UltraLeap Interface.

The Leap Motion Controller 2 [22] (UltraLeap hand tracking, Ultraleap, Bristol, England), seen in Figure 4, features two near-infrared cameras and three light-emitting diodes (LEDs), has a range between 10 cm and 110 cm, a field of view of 160° (H) × 160° (V) and can be used in different orientations, the desktop mode facing up from the table or head-mounted mode, fastened to a virtual reality (VR) headset [23]. Using stereo-vision images, a proprietary algorithm performs the calculation to obtain the position of the joints, being able to accurately measure the angles with high precision. To access the joints’ angles, a software was developed in collaboration with the German Research Center for Artificial Intelligence (DFKI) in Lubeck (Research department AGT), which enables the recording of angles in degrees and saves the data in a CSV file.

### 3.2. Methodology

This study was divided into two parts: the repeatability test and the accuracy test.

#### 3.2.1. Repeatability of the Instruments

To assess the repeatability of the Nuada sensor glove and the UltraLeap Motion, the silicone hand model was used and all the measurements were taken in the same location under the same conditions, ensuring consistency across all instruments. All the calculations were done for all joints of each finger, unless it was not possible according to the instrument. Repeatability was calculated by determining the overall standard deviation for each finger joint.

Nuada sensor glove

The data acquisition process began with testing the repeatability of the Nuada sensor glove under various procedures. These included rotating the hand model in different directions, powering the glove’s wrist unit (watch) on and off, and removing and re-wearing the glove between trials. The goal was to assess the glove’s performance under different conditions and evaluate how system changes, such as recalibration and sensor repositioning, affect its repeatability.

Different orientations procedure: This procedure aimed to assess the glove’s repeatability under stable conditions, without any system changes. The hand model was rotated 90 degrees pointing at four different directions, and each rotation was repeated six consecutive times without recalibrating or adjusting the sensors.

Watch on/off procedure: This step focused on evaluating the impact of calibration errors. For each trial, the same set of movements was performed. Powering the system off and back on after each trial including a recalibration of the magnetometer was performed before each trial. A total of five trials were conducted, allowing for an analysis of how recalibration influenced the consistency of the glove’s measurements.

Glove on/off procedure: This procedure aimed to evaluate the glove’s repeatability when the glove is removed and put back on, assessing the effects of sensor positioning errors. It followed the same sequence as the second procedure but added the step of removing and putting the glove back on before each trial. This was repeated five times to assess the effects of re-wearing, followed by a new calibration on the measurements’ repeatability.

Finally, a diagram was created to compare the standard deviation values from the three procedures: single calibration with varying directions, recalibration, and re-wearing combined with calibration. This diagram helped determine the influence of each factor on the repeatability of the measurements.

UltraLeap

**Figure 5:**
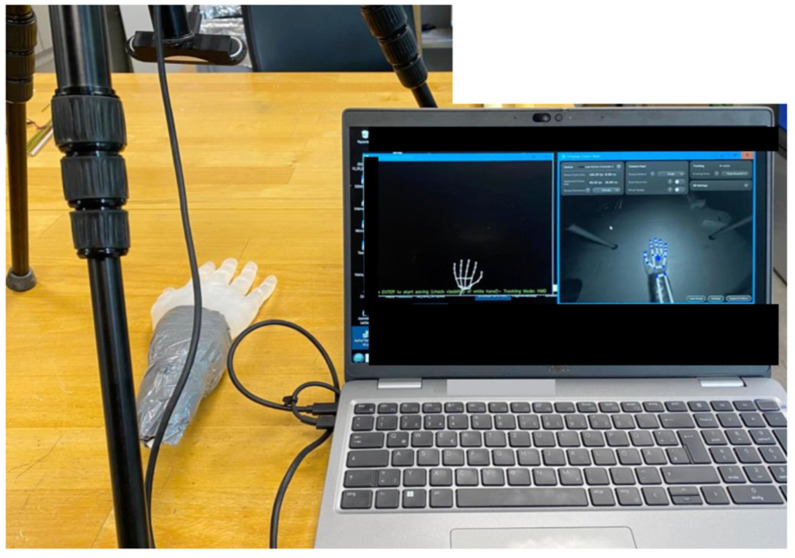
Setup of static positioning using the UltraLeap camera.

To evaluate the repeatability of the UltraLeap camera, a static positioning procedure was developed. Measurements were taken with the hand in a fixed position on the desk due to limitations in the camera’s hand tracking and with the camera mounted on a tripod, capturing the hand from above, as shown in Figure 5. The procedure involved recording the joint angles of the hand model for 90 s. After each recording, the hand model was moved and repositioned in the same static position for subsequent recordings. Five recordings were taken to assess the consistency of the system’s performance over time.

#### 3.2.2. Accuracy of the Instruments

In this study, accuracy refers to the agreement of each instrument’s measurements with the goniometer, which is considered the reference standard in clinical practice, rather than absolute accuracy against a perfectly precise instrument. To evaluate the accuracy of the measuring instruments, the same volunteer’s hand was selected for comparison. An experienced physiotherapist measured the joint flexion and extension angles of the volunteer four times using the goniometer. The same measurements were then taken four times using both the Nuada glove and the UltraLeap system. To assess the agreement between the instruments, the differences and averages of the measurements were calculated and used to create Bland–Altman (BA) plots. These plots are useful for visually comparing the accuracy of each instrument against the goniometer, showing how closely each instrument’s measurements matched the goniometer’s values. The “bias”, represented as the central line in a BA plot, is the average difference between the measurements from the two instruments. This value reveals whether one instrument consistently over- or underestimates the values compared to the other, with a bias close to zero indicating high agreement and a larger bias reflecting greater discrepancy [17,24]. The bias values derived from the comparisons of the goniometer’s measurements with those of the Nuada glove and UltraLeap for each finger joint were analyzed to assess the accuracy of each instrument [17,24]. Finally, the measurement values of the Nuada glove were also compared with those from the UltraLeap system.

## 4. Results

### 4.1. Repeatability

**Figure 6:**
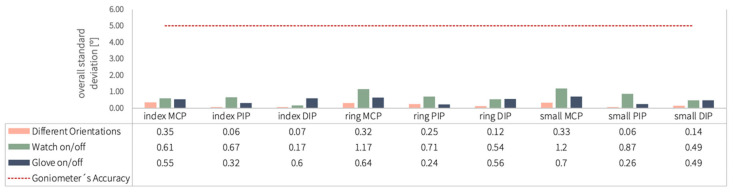
Overall standard deviation for all finger joints within the three procedures conducted with the Nuada glove.

Figure 6 displays the overall standard deviation for each finger joint across the three experimental procedures using the Nuada glove (different directions, watch on/off and glove on/off procedures). Both the thumb and middle finger were excluded after failed calibration of their individual IMU sensors. As calibration is independent per finger, this did not affect the reliability of the remaining measurements. The horizontal red dotted line at five degrees marks the goniometer’s accuracy threshold. Notably, all recorded standard deviations fall below this threshold, with values below two degrees, indicating a high degree of measurement consistency for the tested finger joints.

Additionally, although prior research suggested that cumulative errors would lead to increasing standard deviations across different procedures, this trend was not uniformly observed. For instance, while the standard deviation for the index DIP joint increased as expected across procedures, other joints did not exhibit this rise.

**Figure 7:**
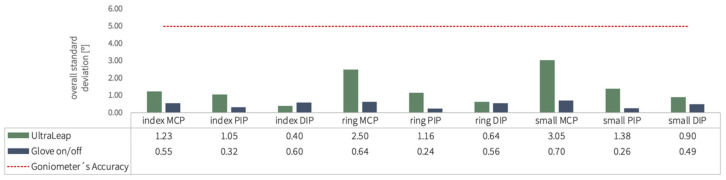
Comparison of the overall standard deviation for all finger joints within the procedure (excluded: thumb and middle finger) conducted with the UltraLeap and the glove on/off procedure with the Nuada glove.

The following graph, Figure 7, displays the UltraLeap results, with all measured standard deviations falling below the five-degree threshold, indicating higher repeatability than the gold standard with all values under 3.5 degrees. The standard deviation values from the glove on/off experiment using the Nuada were also included for comparison. Both instruments exhibited low standard deviations for all joints, with the Nuada glove demonstrating mostly greater repeatability, as shown by its smaller standard deviation values.

These findings confirm that both systems—particularly the Nuada glove—exhibited strong repeatability, remaining well within clinically acceptable limits (<5°). This high consistency supports their suitability for reliable quantitative assessment in rehabilitation contexts.

### 4.2. Accuracy

**Figure 8:**
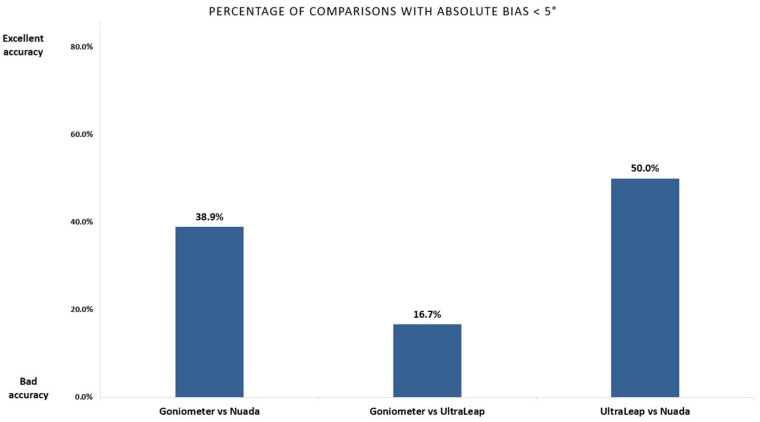
Analysis of the bias value of all comparisons between the Nuada, the UltraLeap and the goniometer.

Figure 8 illustrates the analysis of the “bias”, or average difference in all comparisons made using various instruments, with the goniometer serving as the reference benchmark. This number indicates how closely each instrument’s readings align with the goniometer; the closer the bias is to zero, the more accurate the instrument’s readings are relative to the goniometer.

In this analysis, 40% of the comparisons (seven out of 18) between the Nuada system and the goniometer presented a low bias range, meaning small deviations. This implies that significant deviations were observed for more than half of the comparisons, especially for smaller joints such as the distal and proximal interphalangeal joints.

In contrast, the UltraLeap system showed an even lower percentage of low bias. Only three out of 18 comparisons had average differences below five degrees, showing a higher variability and less agreement with the goniometer.

Interestingly, when the Nuada glove and UltraLeap system were compared directly, there was a higher percentage of their comparisons with an absolute bias below five degrees. So, the two systems are more consistent with each other. This result also implies that the discrepancies between the systems’ measurements are likely due, in large part, to the inherent uncertainty of the goniometer itself.

## 5. Discussion

This study evaluated the repeatability and accuracy of the Nuada glove, an IMU-based sensor glove, and the UltraLeap, an IR camera system, in comparison to traditional instruments like the goniometer, commonly used in physiotherapy to assess hand mobility.

The Nuada glove demonstrated high repeatability, reflected in low standard deviation values across various conditions, including changes in calibration and sensor positioning. While previous studies suggested an increase in the standard deviation of IMU-based measurement systems when the glove is removed after each trial [25], this was not consistently observed in our findings. Variability in results was influenced by the sensitivity of calibration to environmental factors, as well as inconsistencies in how the sensors were positioned. These errors interact in complex ways: when calibration and positioning errors aligned in the same direction, they compounded, leading to cumulative errors; when they opposed each other, they partially canceled out, reducing the overall error. This interplay explains why some procedures did not exhibit the expected increase in standard deviation despite multiple sources of error. Additionally, the “Tare” process, conducted at the beginning of each test, aimed to correct these errors by assuming a flat-hand position as the baseline. However, inaccuracies in this assumed position also contributed to variations in error correction.

Despite these minor discrepancies, the Nuada glove showed robust repeatability, consistent with other studies on IMU-based gloves [10], which reported standard deviations within acceptable ranges for clinical assessment.

The UltraLeap system also showed strong repeatability, with standard deviations consistently below 3.5°, outperforming the goniometer’s accuracy threshold. Prior research supports this finding, showing that the Leap Motion system can reliably track static hand positions with repeatability under two degrees in specific configurations [13].

Comparison with the goniometer showed that most Nuada glove readings differed by more than five degrees, likely due to their distinct measurement methods—goniometers assess individual joints while the Nuada glove measures multiple joints at once. DIP and PIP ranges were measured differently: the goniometer isolates each joint, whereas the glove assesses them during a full fist motion. The UltraLeap device also had limited agreement with the goniometer, possibly because of differences in rotational estimation and measurement perspectives.

The Nuada glove and UltraLeap system aligned better with each other than with the goniometer, suggesting some limitations may stem from the goniometer itself. Calibration inconsistencies affected the Nuada glove’s accuracy, highlighting the need for more reliable sensors and setups.

The IR camera struggled to detect hand features in participants with limited movement, failing to recognize missing fingers in one case. Future improvements should include multiple cameras and enhanced software to address occlusion and better identify amputations or hand irregularities.

## 6. Conclusions

In summary, this study underscores the potential of both the Nuada glove and the IR camera system as reliable tools for clinical hand mobility assessments, showcasing advantages over the traditional goniometer. Unlike the goniometer, which primarily measures static positions, these systems enable continuous measurement, allowing for the tracking of dynamic movements as well. Both systems exhibited superior repeatability and operational efficiency, with the Nuada glove showing slightly higher stability across trials. Although deviations in accuracy relative to the goniometer were observed, the close agreement between the two modern systems suggests that the traditional goniometer may itself introduce measurement uncertainty and may no longer represent the most reliable reference standard. Both instruments further demonstrated greater objectivity, reduced inter-rater variability, and faster measurement acquisition, highlighting their strong potential to streamline clinical workflows.

These findings underscore the growing feasibility of sensor-based and optical technologies for objective, rapid and repeatable hand mobility measurement.

## 7. Limitations and Future Work

During this study, several practical limitations were encountered. First of all, there is no established gold standard for measuring hand and finger movements. The only highly accurate method would be radiological measurement, but this is ethically unacceptable. Therefore, we had to resort to measurement models.

The calibration of the Nuada glove sensors showed slight fluctuations between testing days, requiring recalibration before each session to ensure consistency. For the UltraLeap IR camera, partial finger occlusion occurred when fingers overlapped or when the hand was positioned outside the optimal tracking area. These factors occasionally interrupted continuous data capture and highlighted areas where technical optimization is needed.

Future work will focus on further investigation of the UltraLeap camera due to its simple setup, non-wearable nature and potential for fast clinical application.

Furthermore, the intra- and interrater reliability of the goniometer’s measurements have never been assessed for finger movements. We have now started a trial assessing these aspects.

## Data Availability

The datasets generated and analyzed during the current study are not publicly available due GDPR restrictions but are available from the corresponding author on reasonable re-quest. All data generated or analyzed during this study are included in this published article.

## Abbreviations

The following abbreviations are used in this manuscript:

IMU	Inertial Measurement Unit
IR	InfraRed
ROM	Range of Motion
LMC	Leap Motion Controller
MCP	Metacarpophalangeal
PIP	Proximal Interphalangeal
DIP	Distal Interphalangeal
LED	Light-emitting Diode
VR	Virtual Reality
